# Exploring self-compassion in adults with disabilities: the roles of gender, disability history, and leisure-time physical activity

**DOI:** 10.7717/peerj.19554

**Published:** 2025-06-11

**Authors:** Kashef Zayed, Md Dilsad Ahmed, Mahfoodha Al Kitani, Ali Alyaaribi, Khalifa Al-Jadidi, Ezzeldin Ali, Amin Gaafar, Abdul Rahim Al Droushi, Maryam Alsarmi

**Affiliations:** 1 Sultan Qaboos University, Muscat, Oman; 2 Prince Mohammad Bin Fahd University, Al-Khober, Saudi Arabia

**Keywords:** Adults with disabilities, Self-compassion, Well-being, Disability onset, Leisure-time physical activity

## Abstract

**Background:**

Self-compassion plays a vital role in emotional well-being, especially for individuals with disabilities who often face unique psychological challenges. When people treat themselves with kindness instead of harsh self-criticism, they tend to cope better with difficulties and maintain a more positive outlook. However, self-compassion is not the same for everyone as it can be shaped by many factors like gender, disability history (congenital *vs.* acquired), and participation in leisure-time physical activity. Understanding these influences can help develop better mental health support strategies and more personalized rehabilitation programs.

**Objectives:**

This study explored self-compassion levels among adults with disabilities and examined how gender, disability history, and leisure-time physical activity influence self-compassion.

**Methods:**

A cross-sectional study was conducted with 162 adults with disabilities (85 men and 77 women; average age = 31.3 ± 10.69). Participants completed the Self-Compassion Scale (SCS) and reported how often they engaged in leisure-time physical activity each week.

**Results:**

On average, participants showed moderate self-compassion levels (M = 3.49, SD = 0.58). Among the six components of self-compassion, self-judgment (M = 3.69, SD = 0.90) and over-identification (M = 3.71, SD = 0.92) were the highest, suggesting a tendency toward self-criticism and emotional overwhelm. Self-kindness had the lowest score (M = 3.19, SD = 0.82), indicating that many participants struggled to be compassionate toward themselves. Our further analysis showed that leisure-time physical activity and disability history were significant predictors of self-compassion. People who regularly engaged in sports or exercise and those with congenital disabilities tended to have higher self-compassion levels. Gender, however, was not significantly related to self-compassion. The overall model was significant, F(3, 158) = 10.833, *p* < .001, *R*^2^ = 0.171, explaining 17.1% of the variance in self-compassion.

**Conclusions:**

Many adults with disabilities struggle with self-criticism and emotional distress, but engaging in regular leisure-time physical activity appears to support greater self-compassion. This suggests that making sports and recreation more accessible could help promote emotional resilience in this population. Additionally, the differences observed based on disability history highlight the need for personalized mental health support, as individuals born with a disability may experience self-compassion differently from those who acquired a disability later in life. Future research should further explore these patterns to help improve well-being and psychological support systems for adults with disabilities.

## Introduction

Self-compassion is an important psychological concept that involves being kind and understanding toward oneself during times of struggle or failure, rather than engaging in harsh self-criticism (Neff, 2003). It includes three key aspects: self-kindness, common humanity, and mindfulness. Self-kindness means offering support and care to oneself in difficult moments instead of self-judgment. Common humanity refers to recognizing that challenges and setbacks are a shared part of being human. Mindfulness involves maintaining a balanced perspective on painful emotions, avoiding both ignoring and exaggerating them (Neff, 2003). These elements help individuals face challenges with greater resilience and acceptance.

Research consistently demonstrates the positive effects of self-compassion on various emotional and psychological domains. It fosters positive emotions such as happiness, gratitude, and optimism, which contribute to a more favorable outlook on life (Albertson, Neff & Dill-Shackleford, 2015), Moreover, self-compassion enhances resilience, enabling individuals to effectively manage stress and adversity (Neff & Germer, 2013). These benefits underscore the critical role of self-compassion in promoting psychological well-being and emotional strength.

Self-compassion is strongly associated with life satisfaction, a fundamental measure of overall wellbeing and quality of life (Diener et al., 1985). Extensive research demonstrates a positive relationship between these constructs, showing that individuals with greater self-compassion tend to report higher levels of well-being (Neff, 2011a; Zessin, Dickhäuser & Garbade, 2015).

This connection may arise from self-compassionate individuals’ ability to manage stress and negative emotions, fostering a more positive and optimistic perspective on life (Breines & Chen, 2012). These attributes are especially relevant for individuals with disabilities, as they assist in accepting limitations and coping with challenges. Additionally, self-compassion is associated with heightened motivation and personal growth, which further contribute to life satisfaction and overall well-being (Neff & Germer, 2013).

For individuals with disabilities, self-compassion holds unique significance. They frequently encounter societal barriers and challenges that can negatively impact their psychological health. In recent years, there has been growing attention to the psychological well-being of people with disabilities, with research aiming to identify factors that enhance mental health and self-perception in this population (Alsamiri et al., 2024; Babik & Gardner, 2021).

Self-compassion can be a powerful tool for individuals with disabilities, helping them embrace their limitations while building emotional resilience. Research shows that self-compassion is linked to lower levels of depression and anxiety, providing individuals with effective ways to manage stress and negative emotions, especially when facing disability-related challenges (Neff & Germer, 2013). It also plays a crucial role in adjusting to changes in physical or psychological abilities, making it easier to navigate and overcome obstacles (Leary et al., 2007).

Beyond emotional resilience, self-compassion helps reduce feelings of internalized stigma and encourages a more positive self-image. It also strengthens social connections, as self-compassionate individuals are more likely to seek support and accept help from others, expanding their social networks (Gilbert & Procter, 2006). For individuals with disabilities, this sense of connection is especially valuable, as it fosters a supportive environment that contributes to overall well-being.

Self-compassion also plays a role in promoting physical health by encouraging behaviors that support well-being, such as regular physical activity and adherence to medical treatments (Sirois & Hirsch, 2019). Notably, leisure-time physical activity (LTPA) has been found to enhance psychological well-being by building emotional resilience and reducing stress. The interaction between self-compassion and LTPA is particularly significant, as engaging in LTPA may further cultivate self-compassion by providing individuals with opportunities to care for their bodies and minds (Wong, Chung & Leung, 2021), while self-compassion can motivate consistent participation in LTPA by fostering a kinder, more supportive attitude toward personal health and fitness.

Building on this evidence, the present study investigates the relationship between engagement in LTPA and self-compassion among individuals with disabilities. While existing research highlights the positive effects of physical activity on life satisfaction and quality of life in this population (*e.g.*, Martin Ginis et al., 2010), little attention has been given to its specific influence on self-compassion. Recognizing the established role of LTPA in fostering psychological resilience, this study seeks to explore how LTPA may contribute to self-compassion in individuals with disabilities. By addressing this gap, the research aims to deepen our understanding of the psychological benefits of LTPA and its potential to enhance self-compassion and overall well-being in this population.

## Methods

### Participants

This study included 162 adults with motor disabilities (85 men and 77 women; average age = 31.3 ± 10.69) who volunteered to participate. To make participation as accessible as possible, we used convenience sampling, reaching out through community organizations, disability support groups, and public networks. This approach was chosen because it allowed us to connect with a diverse group of individuals who were both willing and available to take part without placing additional burdens on them.

Trained researchers conducted structured face-to-face surveys, offering support as needed, especially for those with visual impairments or difficulties reading the survey independently. On average, participants took 20 to 30 min to complete the assessment, depending on their individual needs.

The study was reviewed and approved by the Scientific Research Committee of the Department of Physical Education and Sports Sciences, College of Education, which served as the Institutional Review Board (IRB) (Approval Number: PHED/SRC/1862/11/2022). Before participating, all individuals received a clear explanation of the study’s purpose, procedures, potential risks, and benefits. They had the opportunity to ask questions before providing written informed consent.

### Measures

#### Measuring self-compassion

Self-compassion was assessed using the Self-Compassion Scale (SCS), originally developed by Neff (2003). The SCS is a well-established tool that evaluates how individuals respond to personal struggles, capturing both self-compassionate and self-critical tendencies. The full 26-item scale measures six core components of self-compassion: self-kindness (*e.g.*, “I’m kind to myself when I am experiencing suffering”), self-judgment (*e.g.*, “I’m intolerant and impatient toward those aspects of my personality I don’t like”), common humanity (*e.g.*, “When I’m down and out, I remind myself that there are lots of other people in the world feeling like I am”), isolation (*e.g.*, “When I fail at something I tend to feel alone in my failure”), mindfulness (*e.g.*, “When I’m feeling down I try to approach my feelings with curiosity and openness”), and over-identification (*e.g.*, “When something upsets me I get carried away with my feelings”). Participants rated each item on a 5-point Likert scale (1 = rarely to 5 = almost always), with higher scores reflecting greater self-compassion.

The SCS has demonstrated strong psychometric properties, with high internal reliability (Cronbach’s α > 0.86) and robust construct validity (Neff, 2003). It has been widely used to examine the role of self-compassion in well-being, resilience, and mental health. While an Arabic adaptation of the SCS exists (Alabdulaziz et al., 2020), we did not use this version directly. Instead, we modified the scale by considering both the original English version and the available Arabic adaptation, ensuring that the wording was linguistically and culturally appropriate for our study population.

To assess the reliability of the modified version, we conducted a test-retest reliability analysis with 16 disabled adults, who completed the scale twice over a two-week period. The Intraclass Correlation Coefficient (ICC) was 0.84, demonstrating strong reliability and stability within this population. Additionally, the scale exhibited high internal consistency, with a Cronbach’s alpha of 0.882 for the overall measure in our study.

Several shorter versions of the SCS exist, including the 18-item and 17-item versions (Neff et al., 2021), which retain the original six-factor structure but with fewer items, and the 12-item short form (SCS-SF) (Raes et al., 2011), which provides a quicker assessment but has lower reliability for individual subscales. Given the objectives of this study, we selected the full 26-item scale, as it provides the most comprehensive and psychometrically sound assessment of self-compassion.

The SCS is freely available for research, clinical, and educational purposes, as stated by its author, Dr. Kristin Neff. No formal permission is required for its use, provided that the original source is properly cited (self-compassion.org).

#### Measuring leisure-time physical activity

To understand participants’ engagement in leisure-time physical activity (LTPA), we asked them to report how often and for how long they exercised or participated in sports during their free time. Specifically, they provided information on:

• Frequency: How many times per week they engaged in sports or exercise activities.
• Duration: The estimated average time (in minutes) spent on each session.

From these responses, we calculated each participant’s total weekly LTPA by multiplying the number of sessions per week by the average duration per session. The total was expressed in minutes of physical activity per week.

To classify activity levels, we used the physical activity guidelines set by the World Health Organization (WHO):

• Regular engagement: At least 150 min of moderate physical activity per week.
• Moderate engagement: Less than 150 min of physical activity per week.
• No engagement: No participation in any physical activity during leisure time.

These guidelines provide a useful benchmark to assess whether participants met the recommended activity levels for maintaining overall health and well-being.

## Results

This study explored self-compassion levels among adults with disabilities and examined how gender, disability history, and LTPA influenced self-compassion.

### Self-compassion levels among participants

On average, participants had a moderate level of self-compassion (*M* = 3.49, SD = 0.58), based on the SCS, where scores between 2.5 and 3.5 indicate moderate self-compassion (Neff, 2003). However, looking at individual components, distinct patterns emerged.

As shown in Table 1, among the six self-compassion components, Over-Identification (*M* = 3.71, SD = 0.92) and Self-Judgment (*M* = 3.69, SD = 0.90) had the highest scores, suggesting that many participants struggled with self-criticism and tended to dwell on their negative emotions. This means that when facing challenges, they were more likely to judge themselves harshly and get emotionally overwhelmed, rather than responding with balance or self-kindness.

**Table 1:** Mean scores and standard deviations for self-compassion components.

	N	Mean	Std. Deviation
Overall self-compassion	162	3.49	.58
Self-kindness	162	3.19	.82
Self-judgment	162	3.69	.90
Common humanity	162	3.45	.84
Isolation	162	3.60	.90
Mindfulness	162	3.33	.82
Over-identification	162	3.71	.92

Feelings of isolation were also relatively high (*M* = 3.60, SD = 0.90), indicating that many participants felt alone in their experiences and challenges, possibly lacking a sense of connection with others facing similar difficulties.

On the other hand, Self-Kindness had the lowest score (*M* = 3.19, SD = 0.82), meaning that participants found it difficult to treat themselves with warmth and understanding, particularly during difficult times. Mindfulness (*M* = 3.33, SD = 0.82) and Common Humanity (*M* = 3.45, SD = 0.84) fell in the moderate range, suggesting that while participants sometimes maintained emotional balance and recognized shared human struggles, these tendencies were not consistently strong.

### Factors influencing self-compassion

To better understand what shaped self-compassion levels, we conducted a multiple regression analysis to examine whether gender, disability history, and leisure-time physical activity were significant predictors.

As shown in Table 2, the overall model was statistically significant (F(3,158) = 10.83, *p* < .001 and explained 17.1% of the variance in self-compassion (*R*^2^ = 0.171).

**Table 2:** Multiple linear regression predicting total compassion based on LTPA engagement, gender, and disability history.

**Statistics/ Predictor**	**B**	**SE**	β	***t***	***p***	**Model fit values**
Constant	3.165	0.221	–	14.335	<.001	*R*^2^ = 0.171
LTPA engagement	0.001	0.000	0.196	2.660	0.009	Adjusted *R*^2^ = 0.155
Gender	−0.153	0.090	−0.133	−1.712	0.089	F(3, 158) = 10.833
History	0.299	0.091	0.257	3.272	0.001	*p* < .001
Standard error of estimate	–	–	–	–	–	0.532

• LTPA was a significant predictor of self-compassion (B = 0.001, *p* = 0.009). This means that participants who engaged in more frequent physical activity in their free time reported higher self-compassion levels. Physical activity may contribute to greater emotional regulation, reduced stress, and an increased sense of personal empowerment, all of which can help individuals adopt a more compassionate self-view.
• Disability history was also a significant predictor (B = 0.299, *p* = 0.001). Interestingly, individuals with acquired disabilities reported higher self-compassion than those born with a disability. This finding suggests that people who develop a disability later in life may build resilience and self-acceptance over time, whereas those with congenital disabilities may experience lifelong societal and personal challenges that impact their self-compassion levels.
• Gender was not a significant predictor (B = −0.153, *p* = 0.089), meaning that self-compassion levels were similar between men and women in this study. This suggests that, within this sample, other factors played a stronger role in shaping self-compassion than gender alone.

## Discussion

This study explored self-compassion in adults with disabilities, examining how psychological and social factors contribute to self-perceptions and emotional well-being. Findings revealed that participants exhibited a moderate level of self-compassion, consistent with previous research suggesting that individuals with disabilities often face unique psychological and social challenges that influence their self-compassion (Neff, 2011b). This aligns with studies indicating that individuals exposed to external pressures, such as social stigma or accessibility barriers, tend to experience heightened self-judgment and over-identification with their difficulties (Yarnell, Stafford & Neff, 2015). The observed self-compassion levels were also partially consistent with findings from non-disabled adults in the same cultural context (Jansen, Zayed & Kittsteiner, 2020), suggesting that while some aspects of self-compassion may be universal, others are shaped by the specific experiences of living with a disability.

An analysis of self-compassion components revealed that over-identification and self-judgment were the highest-scoring factors, indicating a tendency among participants to engage in harsh self-criticism and emotional over-involvement with negative thoughts. These findings suggest that many individuals in the study struggled to adopt a balanced perspective when facing personal difficulties, reinforcing prior research that links high levels of self-judgment with negative mental health outcomes in populations experiencing chronic adversity (Yarnell, Stafford & Neff, 2015). Feelings of isolation were also relatively high, highlighting the importance of social connection in fostering emotional resilience. In contrast, self-kindness was the lowest-scoring component, indicating that participants found it particularly difficult to respond to themselves with warmth and understanding. This pattern aligns with Gilbert’s model of self-compassion, which suggests that persistent exposure to stressors, such as physical limitations or social marginalization, can hinder the development of self-kindness (Gilbert & Simos, 2022). Mindfulness and common humanity scores fell within the moderate range, suggesting that while participants were sometimes able to maintain emotional balance and recognize shared human experiences, these tendencies were not consistently strong. The findings emphasize the need for interventions that target self-criticism and promote self-kindness, as these may be key areas for improving self-compassion among adults with disabilities.

The regression analysis provided further insight into the factors influencing self-compassion, identifying disability history and leisure-time physical activity as significant predictors. Notably, individuals with acquired disabilities exhibited higher self-compassion than those with congenital disabilities. This finding supports the argument that individuals who develop a disability later in life may cultivate resilience and adaptive coping mechanisms over time, allowing them to integrate self-compassion into their self-perception (Stuntzner & Hartley, 2015). Conversely, those born with a disability may experience prolonged exposure to societal stigma or internalized self-judgment, which could contribute to lower self-compassion levels (Gilbert & Simos, 2022). This highlights the need for early interventions that foster self-compassionate perspectives in individuals with congenital disabilities, particularly in addressing the potential long-term impact of societal attitudes and personal expectations.

The association between leisure-time physical activity and self-compassion further reinforces the psychological benefits of engaging in movement-based activities. Individuals who participated in more frequent physical activity reported higher levels of self-compassion, suggesting that exercise may play a role in enhancing self-awareness, emotional regulation, and overall well-being (Wong, Chung & Leung, 2021). These findings are consistent with previous research indicating that physical activity can facilitate mindfulness, reduce stress, and provide a sense of mastery and empowerment. Given these benefits, promoting accessible and inclusive physical activity opportunities for individuals with disabilities could serve as a viable strategy for enhancing self-compassion and emotional resilience.

Interestingly, gender was not a significant predictor of self-compassion in this sample, suggesting that self-compassion levels were relatively similar between men and women. This is consistent with prior research showing that gender differences in self-compassion are often small or insignificant, particularly in populations with shared life experiences, such as individuals living with a disability (Davies et al., 2021). The lack of gender differences in this study may indicate that other factors, such as disability-related challenges and engagement in leisure activities, play a more central role in shaping self-compassion than gender alone.

The findings of this study have important implications for developing interventions and policies that support self-compassion among individuals with disabilities. Given the positive association between self-compassion and leisure-time physical activity, efforts should be made to ensure that individuals with disabilities have access to inclusive and adaptive sports and recreational programs. Policymakers, rehabilitation centers, and community organizations should prioritize making physical activity opportunities more widely available, as they may serve not only to improve physical health but also to foster psychological well-being (Wong, Chung & Leung, 2021). Additionally, mental health programs should incorporate self-compassion training as part of broader disability support initiatives. Cognitive-behavioral interventions, mindfulness-based self-compassion programs, and structured group workshops may help individuals develop healthier self-perceptions and reduce self-judgment. Given the variation in self-compassion levels based on disability history, psychological interventions should also be tailored to address the distinct needs of individuals with congenital *versus* acquired disabilities (Stuntzner & Hartley, 2015). For those born with disabilities, early-life interventions may be particularly beneficial in fostering resilience and reducing the long-term effects of social stigma.

In addition to psychological interventions, it is important to consider the cultural and social dimensions of self-compassion. In regions such as Oman, where religious teachings and cultural norms play a significant role in shaping self-perception, future research should explore how self-compassion relates to religious and spiritual practices. Incorporating faith-based perspectives into self-compassion programs may enhance their effectiveness in specific cultural contexts by aligning interventions with individuals’ personal beliefs and values (Jensen et al., 2021). Addressing the societal perception of disability through awareness campaigns and inclusive policies may also contribute to a more supportive environment in which self-compassion can thrive.

Although this study provides valuable insights into the psychological experiences of adults with disabilities, certain limitations should be acknowledged. The cross-sectional design prevents conclusions about causality, and self-reported measures may be subject to response biases. Future research should explore these relationships longitudinally to better understand how self-compassion evolves over time in response to life experiences, rehabilitation efforts, or psychological interventions (Neff, 2011b). Additionally, expanding this research to diverse cultural and demographic groups may provide further insights into how self-compassion varies across different contexts.

In conclusion, this study highlights the significant role of leisure-time physical activity and disability history in shaping self-compassion among adults with disabilities. The findings suggest that interventions targeting self-criticism, promoting self-kindness, and increasing access to physical activity could be effective strategies for enhancing emotional resilience in this population. By integrating these insights into rehabilitation programs, community initiatives, and mental health policies, practitioners and policymakers can take meaningful steps toward fostering self-compassion and well-being among individuals with disabilities.

## Limitations

A main limitation of this study is the use of a convenience sample of 162 adults with disabilities. Although this approach made data collection easier, it may not accurately represent the broader population of disabled adults, as participants might differ in self-compassion levels from those who did not participate. Additionally, the study relied on self-reported data for both self-compassion and LTPA engagement, which may introduce biases, such as social desirability bias, potentially affecting the accuracy of the findings.

## Supplemental Information

10.7717/peerj.19554/supp-1 Supplemental Information 1 Dataset Age: A continuous variable representing the participants’ age.
LTPA_total: A continual variable represents the total minutes of engagement in leisure-time physical or sport activities.
Time: Represents the duration (in minutes) spent on LTPA per session.
SC1 to SC26: Items from a self-compassion scale covering aspects such as self-kindness and self-judgment.
Disability: A categorical variable indicating whether the participant has a motor or sensory disability.
History: Refers to the participant’s disability onset (hereditary vs. congenital).
Overall self-compassion: A continuous variable for the total self-compassion score calculated from SC1-SC26: S_Kind, S_Judj, S_Human, Isolation, Mindfulness, Over_Idnt: subscales of self-compassion, representing self-kindness, self-judgment, common humanity, isolation, mindfulness, and over-identification, respectively.

10.7717/peerj.19554/supp-2 Supplemental Information 2 Code List

## Additional Information and Declarations

### Data Availability

The following information was supplied regarding data availability: The data is available in the Supplemental File.
