# Supplementary material for: Exploring self-compassion in adults with disabilities: the roles of gender, disability history, and leisure-time physical activity
Source: PeerJ. 2025 Jun 11;13:e19554. doi: 10.7717/peerj.19554 (PMC12166845; doi:10.7717/peerj.19554)
Supplement: Supplemental Information 2 [file peerj-13-19554-s002.pdf]

Codebook of the categorized variables

Variable	Categories' Description	Code
Gender	Male	1
	Female	2
History	Hereditary	1
	Congenital	2
Time	No engagement at all	0
	One time a week	1
	Two times a week	2
	Three times a week	3
	Four times a week	4
	Five times or more a week	5
